# Neuroprotective Role of a Novel Copper Chelator against A**β**_42_ Induced Neurotoxicity

**DOI:** 10.1155/2013/567128

**Published:** 2013-09-18

**Authors:** Sandeep Kumar Singh, Priti Sinha, L. Mishra, S. Srikrishna

**Affiliations:** ^1^Cell and Neurobiology Laboratory, Department of Biochemistry, Faculty of Science, Banaras Hindu University, Varanas 221005, India; ^2^Department of Chemistry, Faculty of Science, Banaras Hindu University, Varanasi 221 005, India

## Abstract

Alzheimer's disease (AD) is a progressive neurodegenerative disease and associated with the extracellular deposits of amyloid-**β** peptide in hippocampus region. Metal ions like Cu, Fe and Zn are known to associate with the amyloid beta (A**β**) at high concentration and interaction of these ions with soluble and aggregated forms of A**β** peptide help in development of AD. Here we showed Cu mediated neurotoxicity in the eye tissues of transgenic *Drosophila* expressing human amyloid **β** and its rescue through a novel Cu chelator. In this context, we have synthesised and characterized the compound L 2,6-Pyridinedicarboxylic acid, 2,6-bis[2-[(4-carboxyphenyl) methylene] hydrazide] by Mass spectra (MS) and Elemental analysis (EA). The Cu chelation potential of the compound L is tested *in vivo* in *Drosophila*. Oral administration of Copper to the transgenic larvae resulted in severe degeneration in eye tissues, which was rescued by the supplementation of compound L. The levels of anti-oxidant markers like SOD and MDA were measured in compound L treated flies and found a significant rescue (*P* < 0.001). Further rescue of the eye degeneration phenotypes as revealed by SEM affirm the role of copper in A**β** toxicity. Hence, use of compound L, an amidoamine derivative, could be a possible therapeutic measure for A**β** induced neurotoxicity.

## 1. Introduction

Alzheimer's disease (AD) is the most common cause of dementia in the aging population. It is the major neurodegenerative disease of aging brain, mainly associated with the extracellular deposits of amyloid-*β* plaques and intracellular neurofibrillary tangles (NFT) in hippocampus region of the brain. Several studies across the globe show a strong association between loss of metal homeostasis and AD. Consequently, the research community is seriously considering the role of various bimetals and environmental metal toxins in progression and clinical outcomes of Alzheimer's disease and other forms of neurodegenerative disorders. Metals play an important role in Alzheimer's pathology; heavy metals such as lead, cadmium, and mercury especially are highly neurotoxic and have no other biological functions. However, nowadays, people are mainly focusing on biologically important metals such as iron (Fe), zinc (Zn), and copper (Cu) because their imbalance is related to AD. Earlier studies demonstrated that metals like copper and zinc play a critical role in amyloid beta (A*β*) aggregation and neurotoxicity [1–4]. Metal ions, mainly Cu, Fe, and Zn, have been found to colocalize with the amyloid beta (A*β*) in high concentrations, and interaction of these metal ions with soluble/aggregated forms of A*β* peptides has been associated to the development of AD [5]. Among these, copper has attracted the most attention because both the Amyloid beta protein (APP) and amyloid-*β* (A*β*) peptides have significant interaction with the copper.

Location of copper binding domain (CuBD) is found in the N-terminal region of the APP, a Type I transmembrane protein [6]. The CuBD is found in cysteine rich region (between residues 124–189) [7, 8]. His-147, His-151, Tyr-168, and Met-170 are the main amino acid residues of the CuBD, which is involved in the mechanism of Cu coordination and reduction of Cu (II) into Cu (I) [6]. In addition to binding Cu to CuBD of APP, copper is also found to interact with aggregated A*β* and lead to the production of ROS via Fenton's chemistry mechanism [9, 10]. In A*β*, amino acid residues His-6, His-13, His-14, and Tyr-10 are mainly involved in binding with the copper. The copper binding domain present in APP reduces Cu (II) to Cu (I) causes the production of reactive oxygen species, and results in oxidative damage [10, 11].

There are many defence mechanisms that protect the cells from oxidative injuries caused by reactive oxygen species (ROS) like hydroxyl radicals, hydrogen peroxide, superoxide radicals, and singlet oxygen. Superoxide dismutase (SOD) is one of the major antioxidative enzymes which catalyze the conversion of superoxide radical to hydrogen peroxide in the presence of molecular hydrogen [12, 13]. Mainly 3 forms of SOD are present in mammals; copper/zinc SOD (CuZn-SOD, SOD1), which is localized in the cytosol; manganese SOD (Mn-SOD, SOD2), which occurs in the mitochondrial matrix, and SOD3 that is located extracellularly is also a complex of Cu and Zn. Oxidative injury in case of Alzheimer's is well established [14], but the exact role of A*β* peptide and copper ions during this process is controversial [15, 16]. Some people adduced that A*β* toxicity is due to ROS generation in the presence of the A*β*-Cu (II) complex, while others argued that A*β* has antioxidant role. However, Cu plays an important role in the generation of reactive oxygen species [17, 18]. Ongoing research in this area focuses on the prevention of Cu mediated A*β* neurotoxicity and ROS production by Cu chelating therapy, which is an emerging trend in current research. Hence, there is immense need to develop such a suitable copper chelator that could prevent amyloid-*β* aggregation by effectively sequestering extra Cu^2+^ ions. Recently, several groups are focusing on developing such types of new molecules [19–25]. More particularly, in a pioneering work, Storr et al. have developed two carbohydrate-containing compounds,  N,N′-bis[(5-*β*-D-glucopyranosyloxy-2-hydroxy)benzyl]-N,N′-dimethyl-ethane-1,2-diamine (H2GL1)  and  N,N′-bis[(5-**β**-D-glucopyranosyloxy-3-tert-butyl-2-hydroxy)benzyl]-N,N′-dimethyl-ethane-1,2-diamine (H2GL2), that have shown to be promising *in vitro* properties as therapeutic tools against AD [21]. Herein, we designed and synthesized novel compound L, 2, 6-Pyridinedicarboxylic acid, 2,6-bis[2-[(4-carboxyphenyl) methylene] hydrazide], to test the *in vivo* neuroprotective efficacy in a well-established *Drosophila* transgenic model system. 

## 2. Materials and Methods

### 2.1. Chemical Requirements and List of Instruments Used

2,6-Pyridinedicarboxylic acid, hydrazine hydrate, and 4-carboxybenzaldehyde were purchased from Sigma-Aldrich Chem Co., whereas the solvents were purchased from E. Merck and freshly distilled prior to their use. MALDI-TOF Autoflex Speed (Bruker, Germany) was used for MS study.

### 2.2. Synthesis and Characterization of Compound L

The compound L was synthesized in three steps as reported by us earlier [26] starting from 2,6-pyridine dicarboxylic acid (1 mmol, 0.167 g). Its methyl ester was prepared by stirring it in excess methanol in the presence of catalytic amount of concentrated thionyl chloride (SOCl_2_) at room temperature for one day. The ester (1 mmol, 0.171 g) thus isolated was then reacted with aqueous hydrazine hydrate (2.1 mmol, 0.12 mL) in methanol under reflux for 3 h which resulted the production of solid 2, 6-Pyridinedicarboxylic acid, 2,6-bis[2-[(4-carboxyphenyl) methylene] hydrazide]. It (1 mmol, 0.195 g) was finally reacted with 4-formyl-benzoic acid (2 mmol, 0.300 g) in methanol at room temperature. Reaction was monitored using TLC. The product thus obtained was filtered and then purified by repeated recrystallization from hot ethanol. Yield: 85%, elemental analysis calculation for C_23_H_17_N_5_O_6_ (%): C, 60.13; H, 3.70 and N, 15.25. Found (%): C, 60.11; H, 3.67; and N, 15.24. MALDI-TOF/MS, [M + H^+^] = 460.18, [M + Na^+^] = 482.17. See Supplementary Figure (S1) available online at http://dx.doi.org/10.1155/2013/567128. ^1^H NMR (DMSO - *d*_6_, 300 MHz): *δ* (ppm) 13.21 (b, 2H, –COOH), 12.43 (s, 2H, –NH), 8.80 (s, 2H, CH=N), 8.36 (m, 2H, CHpy), 8.34 (m, 1H, CHpy), 8.03 (d, 4H, ArH), and 7.91 (d, 4H, ArH). ^13^C NMR (DMSO - *d*_6_, 300 MHz): *δ*(ppm) 166.98 (C_1_, –COOH), 138.18 (C_2_, ArH), 127.41 (C_4_, ArH), 132.12 (C_5_, ArH), 140.11 (C_6_, –CH=N), 159.70 (C_7_, –C(O)NH), 148.89–148.15 (C_8_, C_9_, Py), and 125.79 (C_10_, Py). IR (KBr pellet, cm^−1^): 3463 (–CONH), 1671 (–COOH), and 1609 (–C=N). 

#### 2.2.1. Crystal Data

C27H35N5O11S2, *M* = 669.74, monoclinic, *a *= 26.7068(15), *b* = 10.1394(4), *c* = 12.4951(6), *α* = 90, *β* = 110.310(6), and *γ* = 90; space group C 2/*c*, *Z* = 4, *V*/Å^3^ = 3173.2(3), reflections collected/unique = 6463/3552 [*R* (int) = 0.0196], and final *R* indices [*I* > 2sigma(*I*)] = *R*1 = 0.0360, *wR*2 = 0.0871.

### 2.3. Synthesis and Characterization of Complex [Cu(L)]**·**2NO_3_

A solution of Cu(NO_3_)_2_·3H2O (0.241 g, 1 mmol) in water (5 mL) was added dropwise to a solution of L (0.459 g, 1 mmol) in DMSO (10 mL). The reaction mixture after stirring for one day at room temperature was left for slow evaporation to get green precipitate. The precipitate was washed with MeOH followed by diethyl ether and then dried in air. Yield: 60%, M.P. >250°C, elemental analysis calc for C_23_H_17_N_7_O_12_Cu (%): C, 39.42; H, 2.42 and N, 14.00. Found (%): C, 39.40; H, 2.43, and N, 14.02. MALDI-TOF/MS [M + H] = 699.17 (S2). IR (KBr pellet, cm^−1^): 3417 (–CONH), 1686 (–COOH), and 1651 (–C=N). UV-vis absorbance: *λ*_max⁡_ (DMSO-water mixture, 10^−5 ^M), nm (*ε*/10^5^ M^−1 ^cm^−1^) 315 (0.66), 352 (0.45), 385 (0.26), and 568 (0.150).

### 2.4. Photophysical Properties of Compound L

UV-vis absorption spectra were recorded on “Jasco V-630” spectrophotometer at 25°C. The absorption titrations of L with copper salt are performed by monitoring the changes in the absorption spectrum of L (10^-5 ^M) in DMSO-water mixture (1 : 9, v/v). The concentration of L is kept constant at 10^-5 ^M, while the concentrations of copper salt are varied within (1–10) ×  10^−6^ M. The absorption of guest molecule is eliminated initially by keeping their equal quantities separately in host L and reference solution. From the absorption data, the intrinsic association constant *K*_*a*_ was determined from a plot of [guest]/(*ε*_*a*_ − *ε*_*f*_) versus [guest] using [27] equation [guest]/(*ε*_*a*_ − *ε*_*f*_) = [guest]/(*ε*_*b*_ − *ε*_*f*_) + [K_*a*_  (*ε*_*b*_−*ε*_*f*_)]^−1^ where [guest] is the concentration of copper salt. The apparent absorption coefficients *ε*_*a*_, *ε*_*f*_, and *ε*_*b*_ correspond to *A*obsd/[L], the extinction coefficient of the free L and extinction coefficient of L in fully bound form, respectively. *K*_*a*_ is given by the ratio of slope to the intercept.

### 2.5. Fly Stocks and Genetics

The *Drosophila* transgenic strain expressing A*β*_42_ under the control of UAS (UAS-A*β* ^H32.12^/CyO) was a generous gift from Dr. M. Konsolaki (Department of Genetics, Rutgers, The State University of New Jersey, USA) and eye specific GAL4 line (ey-GAL4 (w[∗]; P{w[+mC]=UAS-Dab.W}2, P{w[+mC]=GAL4-ninaE.GMR}12/CyO), which directs the expression specifically in eye tissue, used in this study was obtained from Bloomington Stock Center (Bloomington Stock no. 9511), Indiana University, USA. Flies and larvae were reared at 24 ± 1°C on standard *Drosophila* medium containing agar-agar, maize powder, sugar, yeast, nepagin (methyl-p-hydroxybenzoate), and propionic acid. Over expression of the A*β*_42_ transgene under UAS control was achieved by crossing it with ey-GAL4 fly.

### 2.6. Cu/Cu Chelator (Compound L) Treatment

The *UAS- *A*β*_42_ */*ey-GAL4 larvae were cultured in normal food (NF) to achieve eye neurodegeneration phenotypes. The effect of copper on eye degeneration phenotypes was tested by feeding the A*β*_42_ expressing larvae in 500 *μ*M copper nitrate [Cu(NO_3_)_2_] supplemented food. Further, copper (500 *μ*M) along with compound L (250 *μ*M) was tested to see if copper chelation has any effect on eye degeneration in A*β* expressing flies. Wild type Oregon R and undriven *UAS- *A*β*_42_/*UAS- *A*β*_42_ flies were taken as controls in every case (data not shown). The *UAS- *A*β*_42_ */*ey-GAL4 flies from F1 generation with noncurly wings (*n* = 100 in each case) were observed under stereo zoom binocular microscope for scoring eye phenotypes. Data of eye phenotypes was collected in each case of *UAS- *A*β*_42_ */*ey-GAL4 flies cultured on normal food (NF), Cu treated food (Cu food), Compound L treated food, and Cu + compound L supplemented food media, and statistical analysis was done by using one-way ANOVA analysis (PRISM 3 Software).

### 2.7. Superoxide Dismutase (SOD) Assay

Adult flies were homogenized in homogenizing buffer following a method described previously [28]. The homogenate was centrifuged, and SOD activity was estimated as described by Nishikimi et al. [29] with minor modifications as per Singh et al. [28]. One unit of enzyme activity is defined as enzyme concentration required for inhibiting chromogen production (optical density 560 nm) by 50% in 1 min under assay conditions, and the data were expressed as the specific activity in units/min/mg protein.

### 2.8. Assay for Lipid Peroxidation (LPO)

Adult flies were homogenized in homogenizing buffer following a method described previously [28].Malondialdehyde (MDA) content as a measure of LPO was assayed using tetraethoxypropane as an external standard [30]. Lipid peroxide levels were expressed in terms of nmoles MDA formed/h/mg protein.

### 2.9. Scanning Electron Microscopy (SEM) of *Drosophila* Compound Eye

We followed the method of Wolff 2011 [31] with minor modifications for the Scanning Electron Microscopy of compound eyes. About 4–6 representative flies from each group with different treatments were etherized, and heads were detached carefully under the binocular microscope to leave the eyes intact. The decapitated heads with intact eyes were put into 1.5 mL eppendorf tube and fixed overnight in 1.5 mL fixative (0.1 M PBS, 25% glutaraldehyde and dH_2_O), dehydrated in ethanol (once in 25%, 50%, 75%, and 100% ethanol each with 3 hrs and then thrice in absolute ethanol, 15 min each). Tissues were dried by using CPD (critical point drying) for removing any extra moisture present in sample and then analysed by using Scanning Electron Microscope (Hitachi S-3400N). Images were analysed from each group and eye phenotypes were scored.

## 3. Results

### 3.1. Absorption Titration Shows Binding Affinity of Cu^2+^ with Compound L

**Figure 1:**
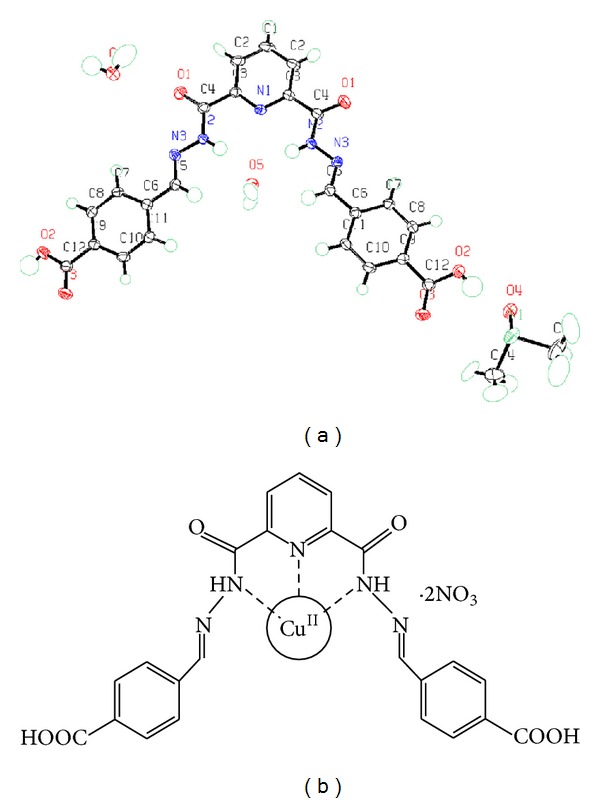
Crystal structure of compound L (a) and structure of compound L with copper binding (b).

The UV-vis titrations with compound L (Figure 1(a)) were carried out in DMSO-water mixture (1 : 9 v/v) solution using standard nitrate salts of Cu^2+^, Zn^2+^, and Ag^+^ at room temperature.

**Figure 2:**
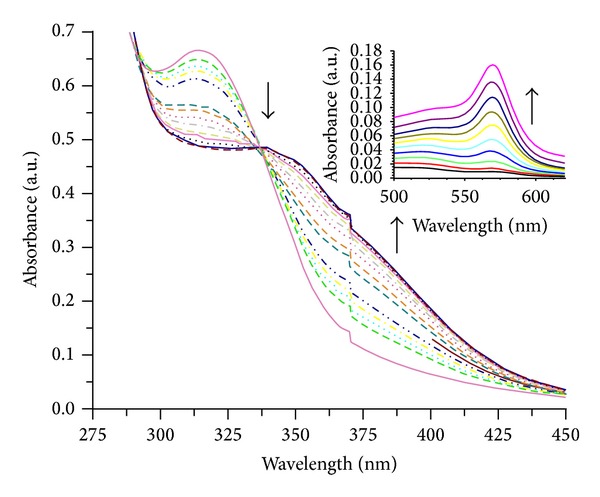
Absorption spectra of compound L = (10^−5 ^M, DMSO-water mixture, 1 : 9 v/v) in the absence and presence of increasing amount of Cu^2+^ = (1–10) × 10^−6^ M (in water) at room temperature. Inset of the d-d region appeared at 569 nm after addition of 1 equivalent of Cu(NO_3_)_2_ solution.

UV-vis spectrum of the solution of L (1.0 × 10^−5 ^M) recorded upon the addition of Cu^2+^, is shown in Figure 2. Upon addition of Cu^2+^ the absorption peak at 315 nm was decreasing, whereas the absorption peak at 352 nm was increasing. The resulting titration revealed an isosbestic point at 290 and 337 nm. The appeared isosbestic point shows that the stable complex (Figure 1(b)) is formed with a definite stoichiometric ratio between L and cation. Interestingly, the addition of other nitrate salts of Zn and Ag did not result in any observable change in the absorption spectrum of L at this wavelength. Colour changes are most probably due to the formation of complex between the amido groups and copper ion. The association constant for copper ion was calculated using equation mentioned in Section 2.4. The value of association constant (*K*_*a*_) for copper ion was found as 1 × 10^6^ binding in 1 : 1 stoichiometry (Job's plot, S3). Electronic spectra for L remained unchanged in the presence of excess (20 mole equivalents) of other nitrate salts of Zn and Ag.

### 3.2. Compound L Ameliorates AD Eye Phenotypes

**Figure 3:**
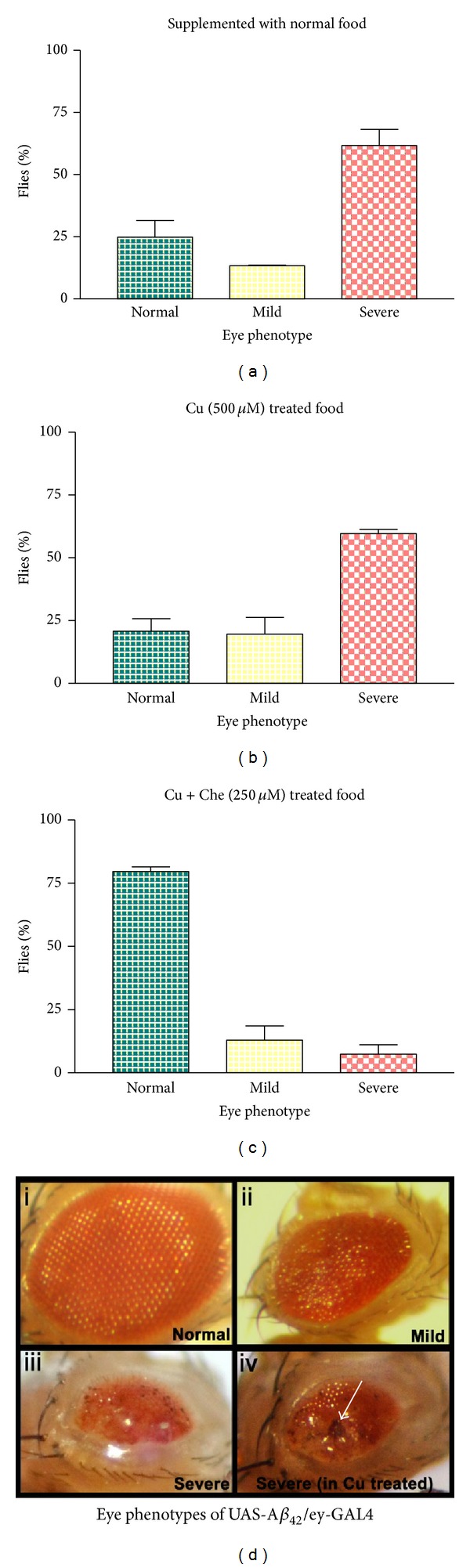
Histogram shows the percentage of flies with normal, mild, and severe eye phenotypes in ey-GAL4 driven UAS-A*β*_42_ flies when grown in normal food (a), copper treated food (b), and Cu + chelator treated food (c). The number of flies (*n* = 100 for (a), (b), and (c)) on *y*-axes is expressed as % of flies against eye phenotype in each case. Different types of eye degeneration phenotypes of *UAS- *A*β*_42_ */*ey-GAL4 like mild ((d) ii) and severe ((d) iii, iv) are shown in panel (d). Note that copper supplemented food resulted in severe ommatidial degeneration as indicated by dark patches in the eye (white arrow in (d) iv).

To determine the effect of copper chelator (compound L) on Cu mediated A*β* toxicity, A*β* expressing larvae were cultured separately in copper (500 *μ*M), chelator (250 *μ*M), and Cu + chelator (250 *μ*M) supplemented food (Figure 3). The flies expressing A*β* were also cultured in normal food (NF), and wild type flies were taken as control in each case. A*β* expressing flies showed mild and severe eye degeneration phenotypes (Figure 3(d) ii and iii, resp.) when cultured in normal food. The severity in eye degeneration was enhanced to several folds in Cu treated flies as compared to untreated flies (compare Figure 3(d) iv with iii), while the percentage of flies showing severe defects was unaltered. The degree of severity was of two types; flies observed form Cu supplemented food showing more severity in eye phenotype with highly degenerative dark patches as compared to flies cultured in normal food (compare Figure 3(d) iv with iii). The statistical significance of these phenotypes was shown in Figure 3. Rescue in severe eye degeneration was found in compound L treated flies at 250 *μ*M concentration (Figure 3(c)), which appears to be the best concentration for copper chelation *in vivo*.

**Figure 4:**
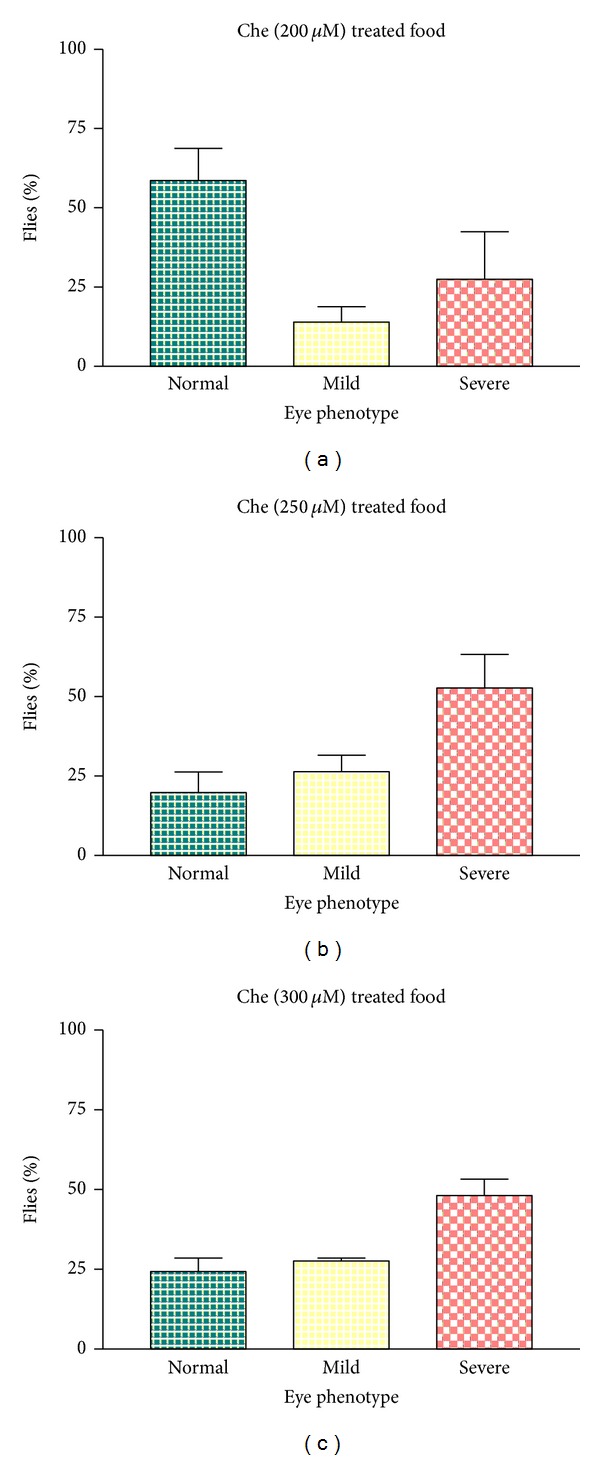
Histogram shows the percentage of flies having normal, mild, and severe rough eye phenotypes in ey-GAL4 driven UAS-A*β*_42_ flies, when grown alone in 200 *μ*M (a), 250 *μ*M (b), and 300 *μ*M (c) of chelator (L) containing food. Note that, at 200 *μ*M, eye degeneration phenotype is rescued as evident by increased number of flies with normal eyes (a). The number of flies in each case is 100.

Compound L (chelator) alone was also tested on *UAS- *A*β*_42_ */*ey-GAL4 flies (without copper supplementation) and found significant rescue only at 200 *μ*M concentration (compare Figure 4(a)with Figure 3(a)). But chelator at 250 and 300 *μ*M concentrations did not show any apparent rescue (Figures 4(b) and 4(c), resp.). There is no lethality found to be associated with these concentrations of chelator. 

### 3.3. Copper Treatment Induces SOD and Lipid Peroxidation

**Figure 5:**
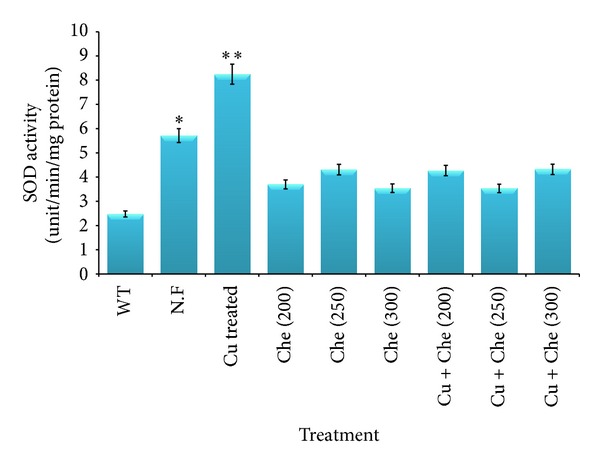
Measurement of Superoxide dismutase (SOD) in wild type flies and A*β* expressing flies treated in normal food as well as Cu and chelator supplemented food. Data represented are mean ± SD of normal and drug treated groups experiments made in three replicates. Significance is ascribed as *P < 0.05 or **P < 0.001 as compared to wild type.

**Figure 6:**
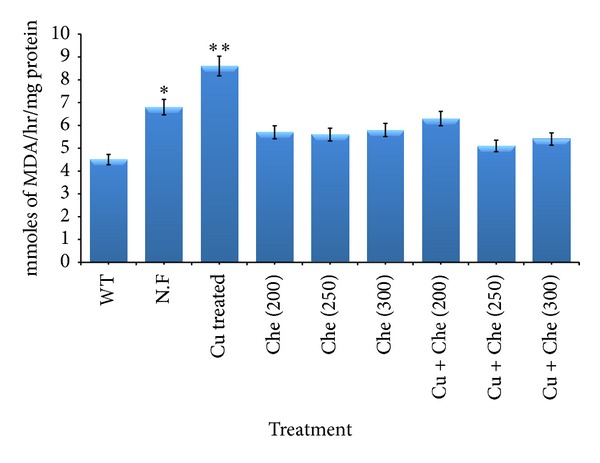
Measurement of malondialdehyde (MDA) content in wild type flies and A*β* expressing flies treated in normal food as well as Cu and chelator supplemented food. Data represented are mean ± SD of normal and drug treated groups experiments made in triplicates. Significance is ascribed as *P < 0.05 or **P < 0.001 as compared to control.

Cu ion enhances the A*β* toxicity following ROS production. The ROS activity in A*β* expressing flies grown in normal food, copper (Cu) treated, chelator (Che) treated, and copper and chelator (Cu + Che) treated food was assessed indirectly by estimating SOD and MDA levels. The effect of copper treatment on antioxidant markers like SOD (Figure 5) and MDA (Figure 6) in A*β*-driven flies suggests increased ROS activity. We observed a significant (*P* < 0.05, *P* < 0.001) increase in the enzyme activity in A*β* flies fed on normal and copper treated food, respectively (Figure 5), and SOD activity was reduced in chelator treated flies, which is comparable to wild type. A similar trend was observed for MDA activity after copper treatment. There was a significant increase of 1.6- and 1.9fold (*P* < 0.05, *P* < 0.001) in MDA activity in flies grown on normal and copper supplemented food, respectively, as compared to wild type (Figure 6).

### 3.4. Scanning Electron Microscopy Shows Rescue in Eye Neurodegeneration

**Figure 7:**
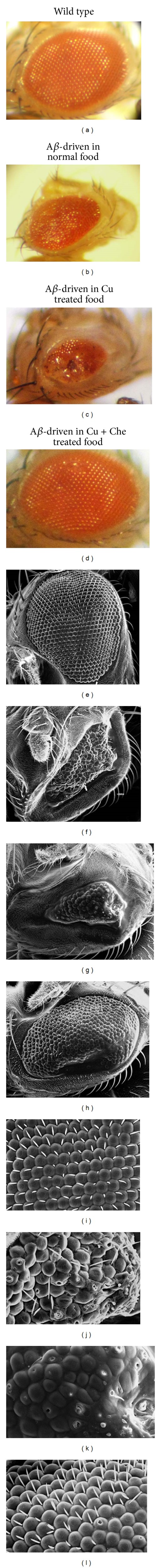
Scanning electron micrographs showing eye degeneration and their rescue after compound (L) treatment ((e)–(l)). Upper panel shows the digital images of compound eyes of wild type (a), A*β* expressing (b), A*β* in presence of copper (c), and A*β* with Cu + chelator (d). There is a reduction in size of eye of A*β* expressing fly grown in normal food ((b), (f)) as compared to the wild type ((a), (e)) and more degeneration can be seen after treatment with Cu (500 *μ*M) alone ((c), (g)). Rescue after the treatment with 250 *μ*M of compound (L) ((d), (h)). Magnification is 230x. Lower panel shows the eye phenotypes of corresponding images of middle panels, respectively, ((e)–(h)) showing very distinct pattern of eye degeneration and ommatidia disruption. Magnification is 700x.

Rescue in degenerative eye is clearly evident in digital microscopy imaging (Figure 7(d)). But in order to clearly visualize the internal morphology of the eye, like structural arrangement of ommatidia and bristles, Scanning Electron Microscopy (SEM) is required. SEM has revealed the recovery of normal eye morphology in chelator treated flies (Figure 7(h)) as compared to flies treated with normal and copper supplemented food (Figures 7(f) and 7(g), resp.). This clearly indicates that compound L may be inhibiting Cu mediated A*β* toxicity which causes eye degeneration. Flies expressing the A*β*_42_ transgene in neurons showed severe eye degeneration when grown in normal food (Figures 7(b) and 7(f)), while food supplemented with Cu ions showed highly degenerative ommatidial morphology with complete loss of bristles and reduction of eye size (Figures 7(c) and 7(g)). However, in wild type control, flies smoothly arranged patterns of ommatidia and bristles are found (Figures 7(a) and 7(e)). 

## 4. Discussion

We have used a *Drosophila* transgenic model of AD to investigate the therapeutic potential of a novel copper chelator, compound L that might be reducing copper mediated A*β* toxicity. Many existing compounds aim to reduce A*β* production by blocking *β* and *γ*-secretases or by stimulating *α*-secretase activity of APP. Recent studies have shown that *β* and *γ*-secretase inhibitors may cause side effects, because they are important for the cleavage of other biologically important molecules [32]. Alternatively, stimulation of the nonamyloidogenic amyloid precursor protein processing is being developed as a potential therapy against AD [33]. Since metals play a very important role in mediated A*β* toxicity, several researchers are working on the development of chelators that can effectively reduce metal toxicity. Further, Cu mediated A*β* toxicity also results in ROS production, so several others are working on this to prevent ROS generation due to Cu induced A*β* aggregation [34]. Therefore, the main aim of this study is to investigate the neuroprotective efficacy of a newly synthesized copper chelator in an *in vivo Drosophila *AD model and to appraise its use as a potential therapeutic agent.

In this work, a novel copper chelator, compound (L), was synthesized and characterized by MS and EA analyses. In order to test its efficacy, compound L was supplemented through diet to the transgenic *Drosophila* expressing human A*β*. We have used UAS/Gal4 system to express A*β*_42_ specifically in eye tissues. The effect of this novel copper chelator in the rescue of severe neurodegenerative eye phenotype was observed by using statistical (Figures 3 and 4) as well as Scanning Electron Microscopic studies (Figure 7). To see the effect of Cu and Cu chelator on a developmentally induced retinal toxicity phenotypes (the severe rough eye phenotype) generated by Cu induced A*β*_42_, we have cultured A*β*_42_ expressing transgenic larvae on normal medium, Cu (500 *μ*M) supplemented medium, Cu + chelator (250 *μ*M), and with chelator alone (200 *μ*M, 250 *μ*M, 300 *μ*M). The A*β*_42_ expressing flies in normal and Cu supplemented food showed mild and severe eye degeneration phenotypes in both the cases (Figures 3(a) and 3(b), resp.). However, the severity in eye degeneration in Cu treated flies is more as compared to the flies grown in normal food though the % number of flies did not vary in both the cases (Figure 3(d)). Interestingly, Cu chelator at 250 *μ*M concentration showed very good rescue against Cu induced severe neurodegeneration phenotype (Figure 3(c)). Further, compound L alone at 200 *μ*M concentration also showed significant rescue against the severe rough eye phenotypes of A*β*_42_ expressing flies (Figure 4(a)). However, 250 *μ*M and 300 *μ*M concentrations did not show such recovery in eye phenotypes (Figures 4(b) and 4(c)), though there is no lethality associated with these doses of chelator. It is unclear why high concentration of chelator did not show better recovery. Perhaps further studies on concentration dependent A*β*_42_ chelator *in vitro* binding assays will shed light on this aspect. We have also checked the effect of copper complex ([Cu(L)]·2NO_3_) on eye phenotypes at the same concentrations used for chelator. But, in this case, there is a lethality observed at both embryonic and early larval stages (data not shown). The lethality is caused due to the presence of high levels of Cu in the complex itself. Similarly, in the SEM analysis, we found that ommatidial irregularity was recovered by the treatment of compound L (Figure 7). We observed the best rescue (~75–80%) of the rough eye phenotype in A*β*_42_ expressing flies when treated with 250 *μ*M of compound (L). Hua et al., showed ameliorating the A*β*-associated toxicity using Cu and Zn chelators [35], thus preventing and/or delaying the progression of AD. Our results show that supplementation with this novel copper chelator reduces copper mediated neurodegeneration by inhibiting A*β* aggregation in *Drosophila* eye. However, the mechanism of action is not clear, and future work should also address the mechanism of action of this novel copper chelator in reducing the copper mediated A*β* toxicity. It is now widely accepted that Cu promotes the A*β* mediated ROS production [17] that causes toxicity to cells. In this context, we have checked some *in vivo* ROS markers like SOD and MDA in treated as well as control flies including wild type and found significant increase in SOD and MDA activities in flies fed on normal as well as copper treated food. This suggests that copper has a role in A*β* toxicity via ROS production. SOD is an antioxidant enzyme and primarily acts to protect oxygen-metabolizing eukaryotic cells from the adverse effects of superoxide ions [36]. Transgenic flies over expressing SOD show a decreased level of oxidative damage and a 33% increase in life span compared to the controls [37]. Generally, SOD activity is observed to be elevated in case of any therapeutic drug, but in our case, we found significantly increased activity of SOD in case of flies fed on normal and copper treated food, and reduction in SOD activity was found after compound L treatment. This observation of elevated SOD activity in copper supplemented flies and reduced activity of SOD in chelator treated flies could be due to Cu and Zn cofactor mediated SOD activity. However, further studies related to SOD and other marker enzymes in this context are required to address this intriguing issue. Thus, our results indicate that this novel copper chelator plays a role in protecting against Cu mediated A*β* aggregation and neurotoxicity in an *in vivo *model system. Our results provide support for the neuroprotective effect of this novel compound L as a potential therapeutic agent for AD.

## 5. Conclusion

In conclusion, we show that the Cu mediated toxicity of A*β* peptides can be reduced through chelation of aggregation-promoting Cu metal ions by suitable chelating agent as developed here (L). In addition, further investigations on the mechanism of action of this novel copper chelator are required. 

## Supplementary Material

MALDI-TOF/MS of adducts as depicted in S1, supported the formation of [M + H^+^] and [M + Na^+^] adducts showing corresponding peaks at 460.18 and 482.17 respectively (S1).
Complex [Cu(L)].2NO_3_ shows MALDI – TOF/MS peak at 699.17 (S2).
Job's plot study shows 1:1 binding mode between ligand L and Cu (NO_3_)_2_ (S3).
