# Supplementary material for: Neuroprotective Role of a Novel Copper Chelator against Aβ 42 Induced Neurotoxicity
Source: Int J Alzheimers Dis. 2013 Sep 18;2013:567128. doi: 10.1155/2013/567128 (PMC3789492; doi:10.1155/2013/567128)
Supplement: Supplementary file 1 — MALDI-TOF/MS of adducts as depicted in S1, supported the formation of [M + H+] and [M + Na+] adducts showing corresponding peaks at 460.18 and 482.17 respectively (S1). Complex [Cu(L)].2NO3 shows MALDI – TOF/MS peak at 699.17 (S2). Job's plot study shows 1:1 binding mode between ligand L and Cu (NO3)2 (S3). [file 567128.f1.doc]

*Supporting information*

*for*

**Neuroprotective role of novel Copper chelator against Aβ_42_ induced Neurotoxicity**

Sandeep Kumar Singh^1^, Priti Sinha^2^, L.Mishra^2^ and S. Srikrishna^1*^

***^1^****Cell and Neurobiology Laboratory, Department of Biochemistry, Faculty of Science, Banaras Hindu University, Varanas-221005, India*

*^2^Department of Chemistry, Faculty of Science, Banaras Hindu University, Varanasi - 221 005, (U.P.) India*

**List of contents**

1. MALDI – TOF MS of ligand (L) in Water – DMSO mixture (9:1, v/v) ….S1
2. MALDI – TOF MS of copper complex in Water – DMSO mixture (9:1, v/v) ….S2

(3) Job’s plot of L in presence of Cu^2+^ ion. ….S3


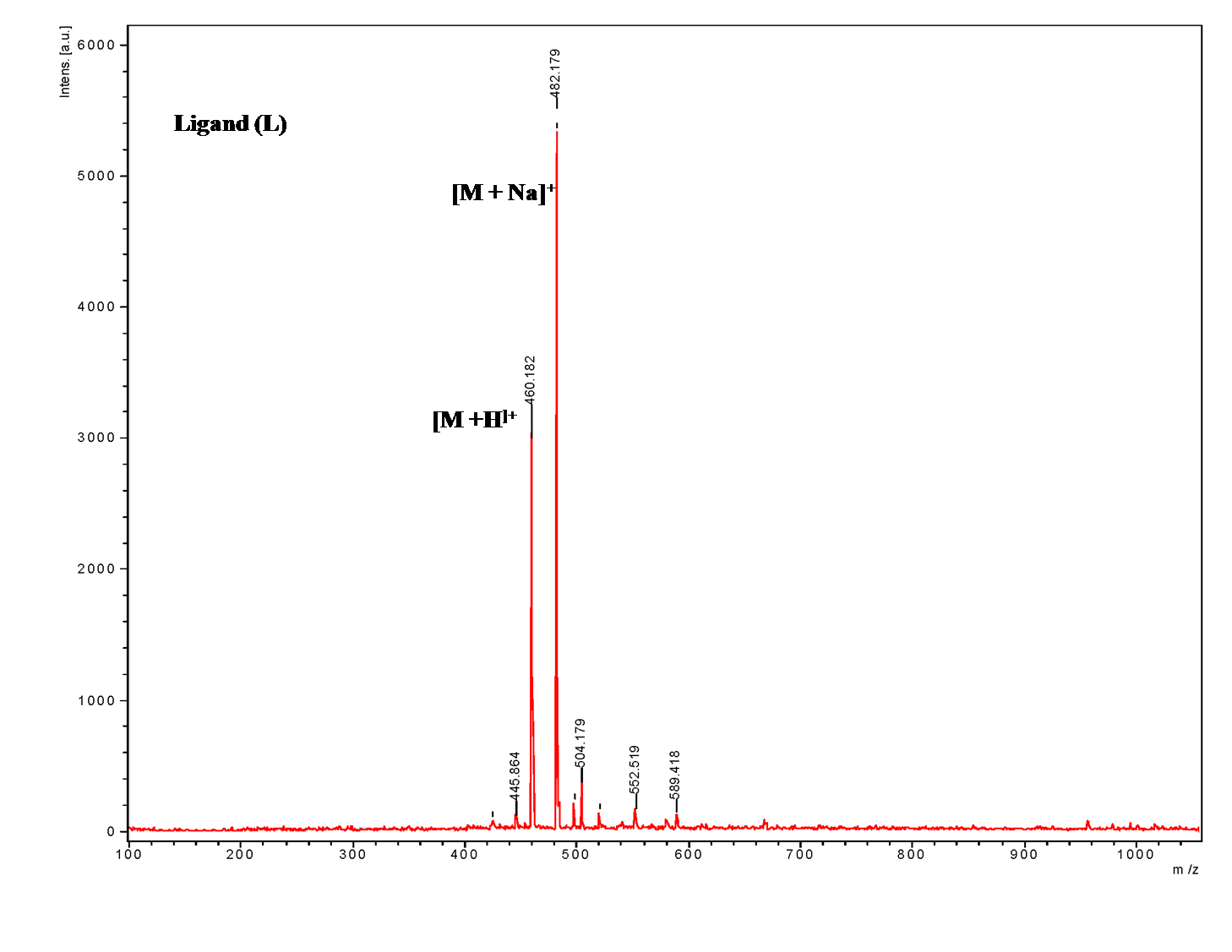


S1. MALDI – TOF MS of ligand (L) in Water – DMSO mixture (9:1, v/v)


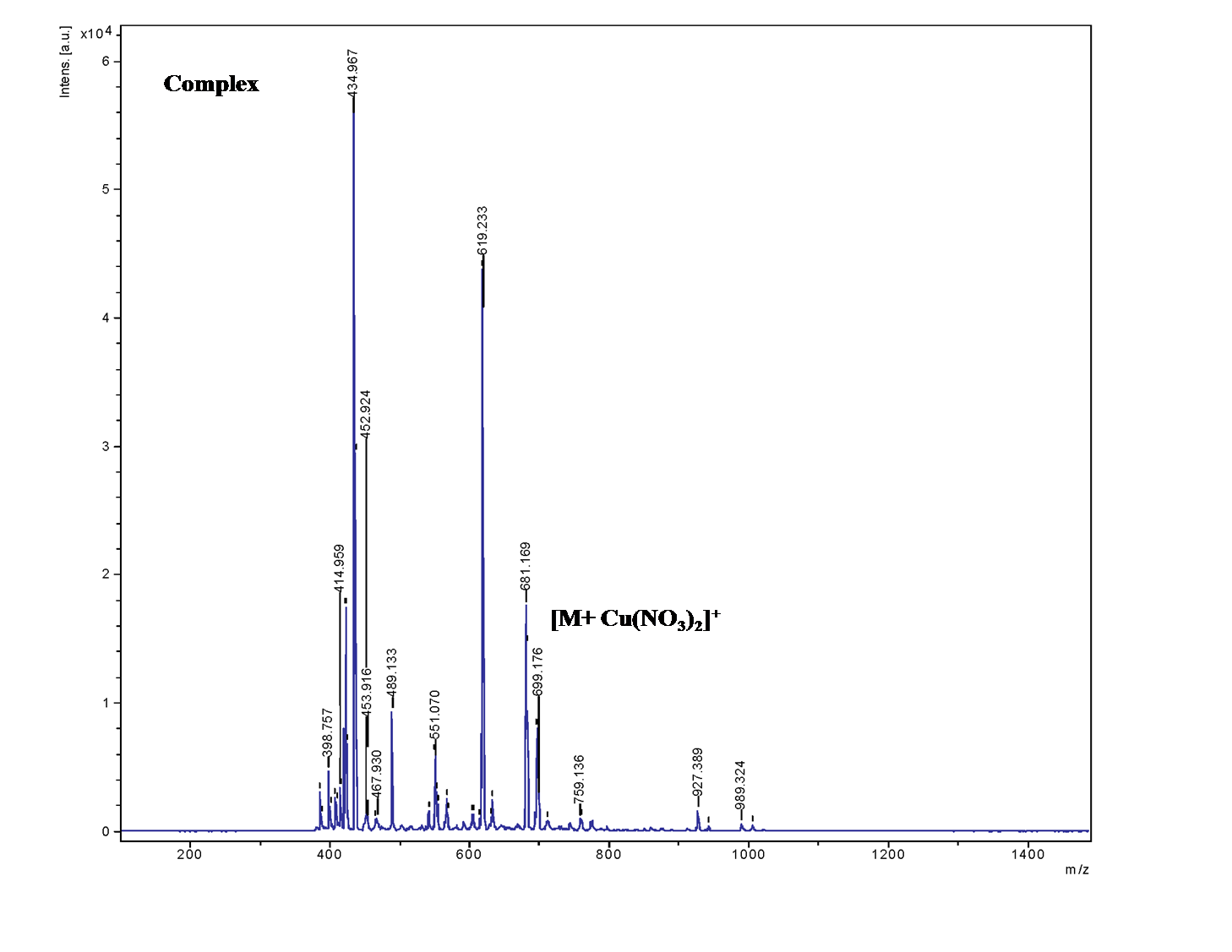


S2. MALDI – TOF MS of copper complex in Water – DMSO mixture (9:1, v/v)


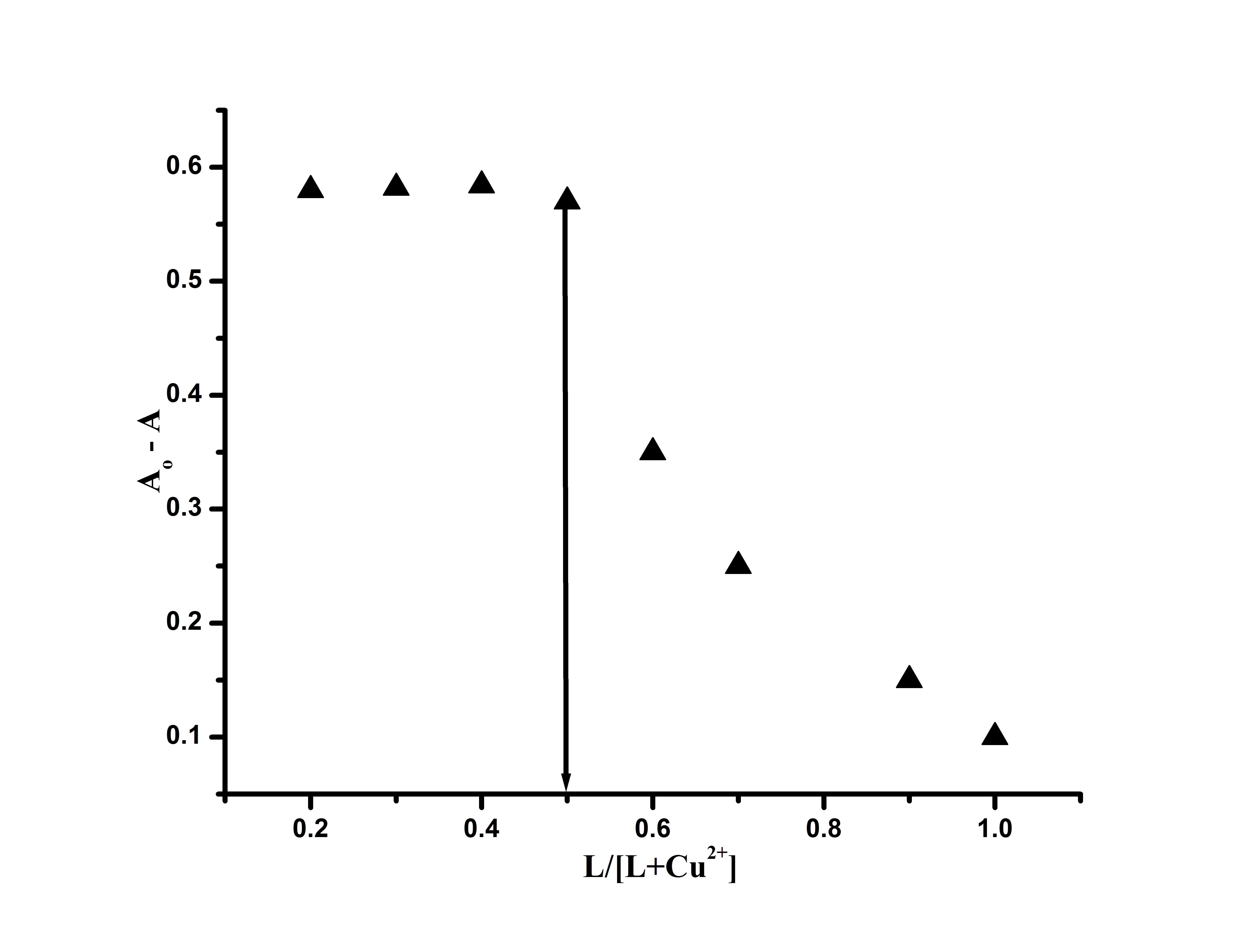


S3. Job’s plot of L in presence of Cu^2+^ ion
